# Supplementary material for: Content-rich biological network constructed by mining PubMed abstracts
Source: BMC Bioinformatics. 2004 Oct 8;5:147. doi: 10.1186/1471-2105-5-147 (PMC528731; doi:10.1186/1471-2105-5-147)
Supplement: Additional File 5 — The original Chilibot query results of the term "long-term potentiation (LTP)" and 22 other terms, limiting the latest references analyzed to the years 1990, 1995, 2000, and 2004. [file 1471-2105-5-147-S5.bz2 › chilibotAdditionalFile5/ltp2000/html/ZIF268.html]

 


**ZIF268** (Input: ZIF268 ) 

---


**Google Searches:** Entire Web  | EDU domain only  | PDF files only

.

**External Links:** OMIM |
LocusLink
| Swissprot
| GeneCards

  
**Maps of ZIF268**

Simple
Complete
graph in
radiant
tree
square
layout.

**New Hypothesis !**

**Synonyms** 

- zif268   [PubMed]

**Synopsis**

- These results suggest that immediate early gene **zif268** may, in part, play a role in the neurotoxicity of NMDA receptor antagonists such as PCP.  Brain Res, 1999 
  [23]

- These results suggest that LTP and spatial training engage a common pathway utilizing an increase in mRNA for raf B, and demonstrate a dissociation between LTP and spatial learning with respect to expression of **zif268** and syntaxin 1B.  Neurosci Lett, 1998 
  [20]

- Together, these results suggest that MAPK, PI 3 kinase  [PI-3K]  and JNK each play a role in the induction of **zif268** gene expression by NGF in PC12D cells.  J Biochem (Tokyo), 1999 
  [20]

- These findings suggest that genetic and activity dependent regulation of **zif268** may influence learning performance.  Hippocampus, 1994 
  [19]

- However, the TrkA isoform containing the variable exon showed significantly higher activation by NT 3, which was detected by stimulation of TrkA autophosphorylation, induction of **ZIF268** transcription, and cellular differentiation.  Proc Natl Acad Sci U S A, 1994 
  [15]

- Non toxic concentrations of anisomycin were found to stimulate these enzyme activities as well as the expression of the early response genes c jun, c fos and **zif268**, and to inhibit NGF induced neurite formation.  Eur J Neurosci, 2000 
  [15]

- From this data we conclude that the synapsin I gene is a target of the **zif268** transcription factor.  J Biol Chem, 1994 
  [14]

- the 50 train stimulus pattern resulted in a robust induction of c fos and c jun mRNA, in addition to **zif268** and junB.  J Neurosci, 1993 
  [14]

- Following each treatment, the expression of cfos, cjun, junB, and **zif268** mRNA changed distinctively and dynamically between 1 and 48 hours.  Synapse, 1998 
  [14]

- Transient increases in intracellular calcium levels resulting from the release of calcium from internal stores alone are NOT sufficient to induce **zif268** mRNA.  J Neurochem, 1997 
  [11]

- From these data, we conclude that the **zif268** egr 1 binding site in the synaptobrevin II promoter is NOT functionally active.  Eur J Biochem, 1996 
  [11]

- Moreover, we demonstrate colocalization of Egr3 and **zif268** mRNAs in neurons of normal and stimulated cortex.  Learn MemLearn Mem, 1996 
  [11]

- Sequencing of the 5 flanking region revealed potential cis acting elements for multiple transcriptional regulatory factors including Sp1, **zif268**, Ets, CREB, and PuF sites.  J Biol Chem, 1997 
  [10]

- These include NGFI A Egr1, **zif268**, NGFI C Egr2, Krox 20 and NGFI B Nurr77 .  Brain Res Mol Brain Res, 1997 
  [10]

- J Neurosci, 1998 
  [10]
